# Effects of COVID-19 Infection on Endothelial Vascular Function

**DOI:** 10.3390/v17030305

**Published:** 2025-02-23

**Authors:** Andreea Mara Munteanu, Daniel Florin Lighezan, Violeta Ariana Nicoras, Patrick Dumitrescu, Olivia-Maria Bodea, Dana Emilia Velimirovici, Gabriela Otiman, Christian Banciu, Daniel-Dumitru Nisulescu

**Affiliations:** 1 Department V, Internal Medicine I—Discipline of Internal Medicine IV, Center of Advanced Research in Cardiology and Hemostasology, “Victor Babes” University of Medicine and Pharmacy, Eftimie Murgu Sq. No. 2, 300041 Timisoara, Romania; 2 Department V, Internal Medicine I—Discipline of Medical Semiology I, Center of Advanced Research in Cardiology and Hemostasology, “Victor Babes” University of Medicine and Pharmacy, Eftimie Murgu Sq. No. 2, 300041 Timisoara, Romania; 3 General Medicine Faculty, “Victor Babes” University of Medicine and Pharmacy, Eftimie Murgu Sq. No. 2, 300041 Timisoara, Romania; 4 Department VI—Cardiology, University Clinic of Internal Medicine and Ambulatory Care, Prevention and Cardiovascular Recovery, “Victor Babes” University of Medicine and Pharmacy, 300041 Timisoara, Romania

**Keywords:** COVID-19, endothelial dysfunction, flow-mediated dilatation, neutrophil to lymphocyte ratio, IL-6

## Abstract

Most studies analyzing data from patients who experienced at least one episode of acute COVID-19 infection have attributed the cascade of immediate and late complications to disruption of the inflammatory system and neutrophil activity in particular. Among the various functions of neutrophils is the release of pro-inflammatory mediators, including interleukin-6 (IL-6). Oxidative stress induced by pro-inflammatory mediators secreted by neutrophils leads to vascular endothelial dysfunction. Neutrophil counts and the neutrophil-to-lymphocyte ratio (NLR) are directly associated with COVID-19 patient survival, with higher values correlating with increased mortality. To assess endothelial dysfunction secondary to COVID-19 infection, we conducted a retrospective study involving two patient cohorts, each comprising 99 participants: one group with a history of COVID-19 infection and another without. The study aimed to demonstrate the presence of endothelial dysfunction in patients with moderate COVID-19 infection using flow-mediated dilatation (FMD) of the brachial artery and to evaluate its correlation with key inflammatory markers (erythrocyte sedimentation rate—ESR, fibrinogen, NLR, IL-6). FMD values were significantly reduced (*p* < 0.0001) in post-COVID-19 patients compared to those without prior infection. ESR (*p* < 0.0001), fibrinogen (*p* < 0.0001), C-reactive protein (CRP) (*p* < 0.0001), leukocyte count (*p* < 0.0001), and granulocyte count (*p* < 0.0001) were inversely correlated with FMD values. Among post-COVID-19 patients, all analyzed parameters demonstrated a statistically significant impact on FMD, with ESR showing the strongest effect, accounting for nearly 63% of the dependency. ANOVA testing confirmed an inverse association between NLR quartiles and FMD, as well as between IL-6 levels and FMD. In conclusion, this study highlights the presence of endothelial dysfunction in post-COVID-19 patients, as assessed by FMD, and demonstrates statistically significant inverse correlations between FMD values, IL-6 levels, and the neutrophil-to-lymphocyte ratio.

## 1. Introduction

The onset of the COVID-19 pandemic, caused by the SARS-CoV-2 virus, was marked by the rapid spread of a novel viral strain of the coronavirus family, first identified in Wuhan, China, in late 2019 [1]. This acute infection triggered a global public health crisis characterized by high transmissibility rates, variable incubation periods, and diverse clinical manifestations, ranging from asymptomatic cases to severe acute respiratory syndrome (SARS), culminating in a significant mortality rate [2].

From the beginning, a direct relationship between SARS-CoV-2 and angiotensin-converting enzyme 2 receptors (ACE2R) was established, with ACE2R being identified as the entry point for the virus into host cells [3]. The virus’s surface spike (S) protein binds to ACE2R on the surface of cells, particularly within pulmonary, cardiac, vascular, and intestinal tissues, facilitating viral entry and infection. This interaction triggers a series of immunological responses that contribute to the varied pathogenesis of COVID-19 [4]. Endothelial cells in these tissues also express ACE2 receptors, creating a direct link between the vascular endothelium, systemic inflammation, and endothelial dysfunction [5]. This interaction compromises endothelial barrier integrity and promotes coagulation, thrombosis, and microvascular damage. Studies on this topic have shown that COVID-19 infection induces endothelial dysfunction through both direct viral effects and virus-mediated activation of inflammatory responses [6].

Although most patients recover their baseline health, a significant subset (10–30%) experience persistent, complex symptoms lasting weeks, months, or even years following the acute phase of the infection [7]. Even the patients having fast recovery after acute COVID-19 infection present later on with cardiac pathologies including arrhythmias, palpitations, chest discomfort, dyspnea and systolic dysfunction and in all these patients endothelial dysfunction is present thus predicting adverse cardiovascular effects [8]. These prolonged effects, termed post-acute sequelae of COVID-19 or long COVID syndrome by the World Health Organization (WHO), involve multiple organ systems and are associated with sustained inflammatory processes, endothelial dysfunction, and immunological dysregulation [9]. Long COVID syndrome includes respiratory, cardiovascular, neurological, and musculoskeletal manifestations, which are often linked to chronic vascular inflammation and impaired vascular homeostasis [8,10,11,12,13]. These conditions result in continued microcirculatory damage and a predisposition to prothrombotic events, exacerbating the severity and duration of post-COVID symptoms [14,15].

In this context, investigating long COVID syndrome is imperative, and evaluating endothelial dysfunction should be a priority in patients presenting with residual symptoms post-COVID-19 infection. The implementation of simple, easily applicable, and non-invasive methods for assessing inflammatory biomarkers and endothelial function is crucial for risk stratification and the early detection of potential cardiovascular complications in these patients [5].

Flow-mediated dilatation (FMD), evaluated using ultrasonography, is currently a non-invasive method recommended by clinical guidelines for assessing endothelial function. FMD measures endothelium-dependent vasodilation of the brachial artery during reactive hyperemia as a response to shear stress induced by ischemia, leading to nitric oxide release from the endothelium and subsequent vasodilation [16].

Recent studies have demonstrated the persistence of endothelial dysfunction in long COVID syndrome, reflected by reduced FMD [17]. However, these studies have not established direct causal relationships with inflammatory biomarkers, hindering the development of targeted therapies aimed at reducing inflammation and improving vascular endothelial function to mitigate long-term cardiovascular risks.

## 2. Materials and Methods

This retrospective study was conducted in the Internal Medicine Clinic of CFR Clinical Hospital in Timișoara between 1 January 2022 and 1 November 2024. The hospital, recognized for its expertise in managing COVID-19 patients, provided care during both the acute phase of infection (moderate to severe cases not requiring ventilatory support) and the post-COVID-19 phase. Ethical approval was obtained from the Ethics Committee of the Clinic Hospital of Transport Ministry, Timisoara, Romania (reg. nr. 12766/2 December 2024).

The aim of this study was to evaluate the response of the vascular endothelium to COVID-19 infection and to assess its correlation with the magnitude of the inflammatory syndrome during the acute COVID-19 phase, regardless of the presence or absence of endothelial dysfunction.

Endothelial dysfunction was assessed using FMD measurements of the left and right brachial arteries. A Fujifilm Diagnostic Ultrasound System Arietta 65 was utilized to measure the maximal transverse diameters of the arteries before (Di) and 1 min after cuff release (Df). Longitudinal axis imaging was performed. Arterial occlusion was achieved by inflating a sphygmomanometer cuff placed 5 cm above the antecubital fossa to 50 mmHg above the pressure at which the radial artery pulse ceased. FMD was calculated using the formula (Df − Di)/Di] × 100. The sphygmomanometer cuff was inflated to 50 mmHg above the pressure at which the radial artery pulse ceased for a duration of 5 min to ensure proper occlusion. Recommendations for patients before measurements: Patients were instructed to avoid consuming caffeine, nicotine, or engaging in strenuous physical activity for at least 12 h prior to the measurement. Additionally, patients fasted for at least 8 h to minimize any potential confounding effects. Conditions during measurements: Measurements were performed in a temperature-controlled room (22–24 °C) after a 10 min rest period, with the patient being placed in a supine position to ensure consistency and reduce external influences on vascular function. Endothelial dysfunction was defined as having an FMD value below 11.1% [18].

The study analyzed 198 patients divided into two groups: Group 1 included 99 patients still experiencing symptoms three months post-COVID-19 infection (long COVID syndrome), and Group 2 included 99 patients without a history of COVID-19. Inclusion in the post-COVID-19 group was based on consecutive outpatient visits until the required sample size was achieved. The control group was similarly selected and matched for age and comorbidities on a 1:1 basis to minimize confounders and ensure reproducibility of the data.

The inclusion criteria for Group 1 involved documented COVID-19 infection within the previous 3–6 months, verified by official laboratory records or physician-documented test results. Group 2 consisted of patients without a history of COVID-19.

Exclusion criteria for both groups included the presence of cardiovascular diseases other than hypertension (HTN), diabetes mellitus, lipid-lowering therapy, Grade II–III obesity, metabolic syndrome, history of stroke, treatment for hyperuricemia, and rheumatic diseases. Hypertension (HTN), diabetes mellitus, lipid-lowering therapy, grade II–III obesity, metabolic syndrome, history of stroke, treatment for hyperuricemia, and rheumatic diseases were kept among the elements that do not exclude the inclusion of patients based on several expectations. These conditions are prevalent in the general population and particularly common in patients at risk of or recovering from COVID-19. Including them allows the study to reflect a real-world patient population and enhances the generalizability of the findings. To minimize their impact as confounding factors, the control group was carefully matched for these conditions on a 1:1 basis with the post-COVID-19 group. This ensured that any observed differences in endothelial dysfunction could be more reliably attributed to the effects of COVID-19 rather than pre-existing comorbidities. Excluding these conditions would have significantly narrowed the scope of the study. As many of these comorbidities are closely linked to cardiovascular health and endothelial function, their inclusion provides insights into how COVID-19 might exacerbate endothelial dysfunction in individuals already at risk of cardiovascular events.

The presence of COVID-19 infection was determined by a positive test recorded in the official records by laboratories that performed testing for COVID-19 infections or by doctors who conducted testing for COVID-19 infection.

The data collected included demographic characteristics (age, sex, height, weight), smoking behavior, complete blood counts, erythrocyte sedimentation rate (ESR), fibrinogen, FMD, C-reactive protein (CRP), D-dimer, ferritin, interleukin-6 (IL-6), lactate dehydrogenase (LDH), and HTN status.

HTN was defined as ongoing antihypertensive therapy or systolic/diastolic blood pressure exceeding 140/90 mmHg during hospitalization, as per the European Society of Cardiology guidelines [19].

Metabolic syndrome was diagnosed based on International Diabetes Federation criteria, requiring central obesity or a raised abdominal circumference alongside at least two additional criteria: systolic BP ≥ 130 mmHg or diastolic BP ≥ 85 mmHg, serum triglycerides ≥ 150 mg/dL (or treatment for hypertriglyceridemia), HDL cholesterol ≤ 40 mg/dL for men or ≤50 mg/dL for women, and fasting plasma glucose ≥ 100 mg/dL or previous diagnosis of type 2 diabetes [20,21]. Nutritional status was categorized using body mass index (BMI): overweight (BMI 25.0–29.9) and obese (BMI ≥ 30).

A statistical analysis was performed using MedCalc version 23.0.9 software for Windows. A *p*-value of <0.05 was considered significant. Descriptive statistics were used to summarize data, including means, standard deviations, ranges, medians, and interquartile ranges for continuous variables, as well as counts with percentages for categorical variables. Quartiles were calculated using a Tukey’s method. Independent samples t-tests were employed to compare continuous variables, while categorical variables were analyzed using the Chi-squared test or Fisher’s exact test. Logistic regression was used to evaluate the odds of endothelial dysfunction. Results are presented in tables and figures where appropriate.

## 3. Results

The mean age of patients included in the study was 63.78 years (95% CI: 61.69–66.38) for Group 1 (post-COVID-19) and 62.65 years (95% CI: 60.29–65.01) for Group 2 (control), with no statistically significant difference between the two groups (*p* = 0.5227; 95% CI: −4.61–2.35).

**Figure 1:**
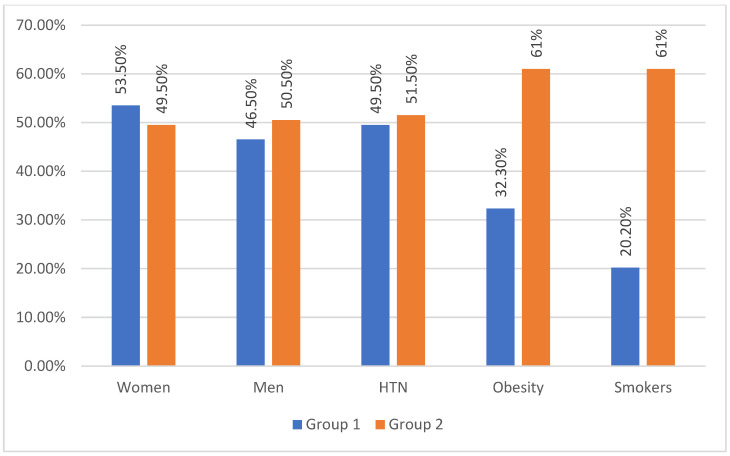
Group characterization according to gender, presence of hypertension, obesity, and active smoking (HTN—HTN-hypertension). Group 1—post-CIVID-19 patients; Group 2—patients without a COVID-19 history.

Regarding sex distribution (*p* = 0.5705), presence of arterial hypertension (HTN) (*p* = 0.7767), obesity (*p* = 0.6451), and smoking status (*p* = 0.3139), no significant differences were observed between the groups. These characteristics are detailed in Figure 1.

The mean values of the analyzed parameters for comparison between the two groups are summarized in Table 1.

FMD values were significantly lower (*p* < 0.0001) in patients post-COVID-19 compared to those without prior infection, indicating marked endothelial dysfunction in the post-COVID-19 group. The factors correlated with reduced FMD values are detailed in Table 2 for patients in Group 1 and Table 3 for patients in Group 2.

An analysis of the data presented in Table 2 and Table 3 indicates that the majority of factors investigated for their association with endothelial dysfunction, defined as FMD values below 11.1%, demonstrated statistically significant correlations. Exceptions included platelet count (*p* = 0.7851), hemoglobin levels (*p* = 0.0884), and hematocrit levels (*p* = 0.1600). Notably, platelet count and hematocrit levels also showed no significant associations in patients without a history of COVID-19.

Given the large number of factors correlated with FMD values below 11.1%, we aimed to assess the degree of dependency of FMD impairment on each factor through a simple linear regression analysis. The dependency data are summarized in Table 4 for Group 1 (post-COVID-19 patients) and Table 5 for Group 2 (patients without COVID-19 history).

For Group 1 (post-COVID-19 patients), all analyzed parameters demonstrated a statistically significant impact on the dependent variable, FMD. Among these, the highest degree of dependency was observed for the ESR, which accounted for approximately 63% of the variation in FMD. Fibrinogen levels explained approximately 50% of the variation in FMD, highlighting its substantial role. About 40% of the variation in FMD was attributed to lung damage, as quantified by the extent of “ground-glass” opacities observed in chest computed tomography (CT) scans and CRP levels. D-dimer, ferritin, and IL-6 levels influenced approximately 30% of the FMD variation. Furthermore, granulocyte count and the neutrophil-to-lymphocyte ratio (NLR) accounted for 23% of the FMD variation. All analyzed parameters were found to have a significant effect on the dependent variable, FMD, reinforcing the multifactorial nature of endothelial dysfunction in post-COVID-19 patients.

In patients without a history of COVID-19 (Group 2), statistically significant associations with FMD were observed only for ESR, fibrinogen, CRP, and D-dimer levels. Among these, ESR demonstrated the highest degree of dependency, although its impact was somewhat lower compared to the post-COVID-19 group. CRP values appeared to have a similar influence on endothelial dysfunction in both groups.

To evaluate further the dependency of FMD on various factors in Group 1, particularly identifying those with a significant impact, a multiple linear regression model was constructed. The results are summarized in Table 6.

This model explained approximately 65% of the variation in FMD values. Among the included factors, ESR and the neutrophil-to-lymphocyte ratio (NLR) were identified as having a statistically significant effect on FMD variation. Other factors included in the model did not demonstrate a significant impact.

**Figure 2:**
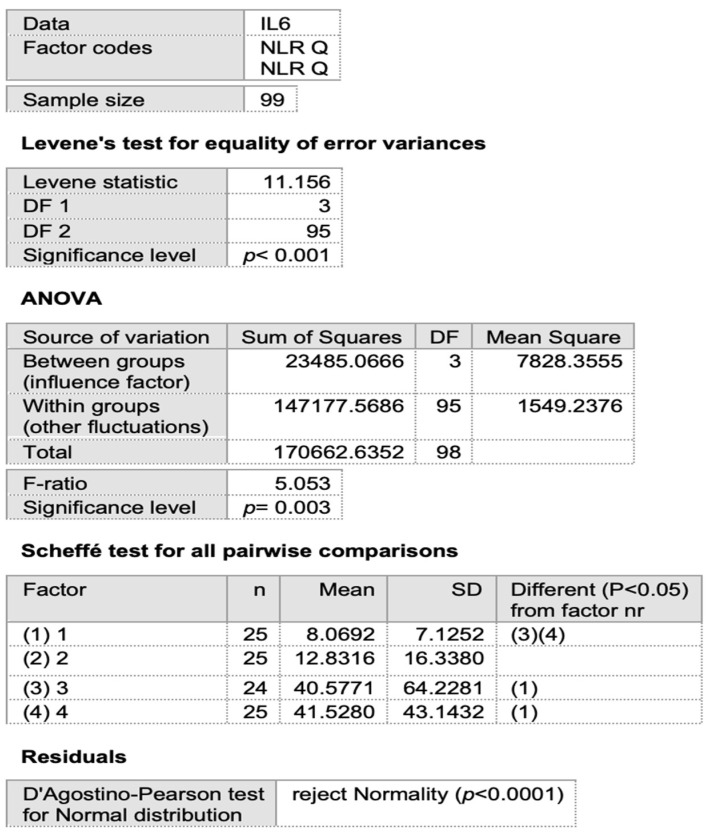
ANOVA test for quartiles of the IL-6 and neutrophil-to-lymphocyte ratio (NLR).

The negative correlation observed between the ESR and NLR in regard to FMD in our model suggests that any increase in these parameters is associated with a decrease in FMD among Group 1 (post-COVID-19) patients. To further explore the relationship between the NLR and FMD, we divided Group 1 into quartiles based on NLR values and performed an ANOVA test. This analysis revealed a statistically significant difference in FMD values across the NLR quartiles (F (3,95) = 9.712, *p* < 0.001). Post hoc Scheffé pairwise comparisons identified significant differences between specific quartiles: Quartile 1 (Q1): n = 25, mean = 8.2560, SD = 0.6777, significantly differs from Q3 and Q4. Quartile 2 (Q2): n = 25, mean = 8.1360, SD = 0.8751, also significantly differs from Q3 and Q4. Quartile 3 (Q3): n = 24, mean = 7.1417, SD = 0.8005, significantly differs from Q1 and Q2. Quartile 4 (Q4): n = 25, mean = 7.3520, SD = 1.1288, significantly differs from Q1 and Q2. The ANOVA results indicate significant differences in FMD mean values based on NLR quartiles (Figure 2, Table 7). Additionally, a statistically significant difference exists between the first two NLR quartiles (Q1 and Q2) and the latter two (Q3 and Q4), with higher FMD values observed in the lower NLR quartiles (Q1 and Q2).

This analysis suggests a clear relationship between the NLR and FMD, where lower NLR values are associated with higher FMD values. The Scheffé test further confirms these differences, highlighting the quartile pairs that differ significantly.

The first two quartiles (Q1 and Q2) exhibited significantly lower mean IL-6 levels compared to the last two quartiles (Q3 and Q4). This finding suggests a relationship between the NLR and IL-6, where lower NLR values are associated with lower IL-6 levels. A post hoc Scheffé analysis confirmed these differences, identifying the quartile pairs with significant variations.

To further investigate, the correlation between IL-6 levels (categorized into two subgroups: Sample 1—elevated IL-6 values; Sample 2—normal IL-6 values) and FMD was evaluated in the post-COVID-19 patient group. Patients with normal IL-6 levels demonstrated significantly higher FMD values (mean = 35.4654, SD = 46.7241) compared to those with elevated IL-6 levels (mean = 2.9153, SD = 1.3840), with *p* < 0.0001 and a 95% CI of 21.3142 to 43.7857.

Additionally, NLR values were compared between the same IL-6 subgroups. Elevated IL-6 levels were associated with lower NLR values (mean value = 2.9709, SD = 1.9432), while normal IL-6 levels corresponded to higher NLR values (mean value = 5.5132, SD = 5.9508), with *p* = 0.0020 and a 95% CI of 0.9546 to 4.1300.

In conclusion, FMD values demonstrate significant correlations with both IL-6 levels and the neutrophil-to-lymphocyte ratio (NLR). Elevated IL-6 levels and higher NLR values are associated with reduced FMD, suggesting their role in endothelial dysfunction among post-COVID-19 patients.

## 4. Discussion

Most published studies analyzing data from patients who experienced at least one episode of acute COVID-19 infection have attributed the cascade of immediate and long-term complications to the dysregulation of the inflammatory system, particularly neutrophil function and dysfunction [22]. Apart from all typical symptoms, there is evidence of immediate as well as long-term atypical symptoms in COVID-19-infected patients [8,13,23]. Among the various roles of neutrophils is the release of pro-inflammatory mediators, including interleukin-6 (IL-6) [22]. Oxidative stress induced by pro-inflammatory mediators secreted by neutrophils contributes to vascular endothelial dysfunction [22]. Elevated IL-6 levels have also been associated with long COVID-19 syndrome [24].

To evaluate the impact of post-COVID-19 inflammation on the vascular endothelium, we designed our cohorts to minimize confounders. Patients with known dyslipidemia, diabetes, or obesity were excluded due to the paradoxical effects of these conditions on the cardiovascular system [25]. Previous studies have also linked endothelial dysfunction in obese patients to prior episodes of COVID-19 infection [26].

The neutrophil-to-lymphocyte ratio (NLR) has garnered significant attention recently due to its minimal additional cost, as it can be derived from a standard complete blood count without requiring specialized tests or equipment.

Elevated NLR values are associated with increased mortality and complications, including arrhythmias, following procedures or surgeries [27].

Higher neutrophil counts in COVID-19-positive patients have been correlated with increased endothelial dysfunction (ED) incidence [26]. However, some studies assessed leukogram parameters long after COVID-19 infection, potentially confounding correlations with post-COVID-19 FMD.

In our study, granulocyte and leukocyte counts were significantly higher in patients without a history of COVID-19 in comparison to patients with COVID-19 history. This could be attributed to the fact that leukogram parameters in the COVID-19 cohort were assessed during hospitalization for acute infection, aligning with findings of elevated neutrophil counts and NLRs in COVID-19 patients. Elevated neutrophil counts and NLRs have been strongly associated with increased mortality in COVID-19 patients [28] and independently predict disease severity [22].

In our cohort, low NLR values were associated with normal endothelial function (FMD > 11%), while elevated NLR values were associated with endothelial dysfunction, consistent with prior observations linking high neutrophil counts to ED in COVID-19 patients.

Elevated neutrophil counts are also associated with lymphocyte suppression, a common feature in severe COVID-19 cases [29]. This suppressive mechanism could partly explain the direct relationship between increased NLRs and disease severity. Despite relatively moderate increases in circulating granulocytes in our study population, likely due to the inclusion of patients with moderate COVID-19, NLRs remained elevated compared to the non-COVID-19 group. This increase was primarily driven by a more pronounced decrease in lymphocyte counts rather than a significant increase in neutrophil counts.

Reduced FMD values, indicative of endothelial dysfunction, have been inversely correlated with higher IL-6 levels in patients with severe COVID-19 requiring intensive care unit (ICU) admission [30]. In our study, endothelial dysfunction, as evidenced by reduced FMD, was inversely correlated with serum IL-6 levels in patients with moderate COVID-19. Higher IL-6 levels were associated with lower FMD. Other inflammatory markers, such as the ESR, also demonstrated similar correlations, although the NLR and ESR played a more significant role in predicting this association. Endothelial dysfunction was also observed in patients without a history of COVID-19, following a similar pattern for all analyzed inflammatory markers, underscoring the broader negative impact of systemic inflammation on vascular endothelial function. Smoking plays an important role in endothelial dysfunction, the cessation of which can improve health outcomes. Various parameters like psychological stress, peer smoking, and social factors all can lead to increased consumption of tobacco causing serious long-term cardiovascular health issues [31,32].

Given the observed link between endothelial dysfunction and inflammation in post-COVID-19 patients, targeted therapies aimed at reducing inflammation and improving vascular endothelial function could play a crucial role in mitigating long-term cardiovascular risks. Strategies such as the use of statins, which have anti-inflammatory and endothelial-protective effects, may be beneficial in reducing vascular inflammation [33]. Similarly, angiotensin-converting enzyme inhibitors (ACEIs) and angiotensin receptor blockers (ARBs) could improve endothelial function by modulating the renin–angiotensin–aldosterone system [34]. Furthermore, antioxidant therapies, including supplementation with vitamins C and E, have shown promise in regard to reducing oxidative stress and enhancing endothelial nitric oxide bioavailability [35]. Emerging therapies, such as monoclonal antibodies targeting pro-inflammatory cytokines like IL-6, may also offer novel avenues for addressing inflammation-driven endothelial dysfunction [36]. Integrating these therapies into clinical practice could provide effective strategies for preventing adverse cardiovascular outcomes in post-COVID-19 patients [37].

Study Limitations. As a retrospective study, one of the primary limitations is the lack of routine evaluation of biomarkers such as lactate dehydrogenase (LDH), ferritin, and IL-6 in patients without chronic cardiovascular conditions, e.g., coronary syndrome or heart failure. These biomarkers were measured exclusively in COVID-19 patients to assess infection severity. By addressing the inflammatory and endothelial dysfunction mechanisms in COVID-19 and non-COVID-19 populations, this study highlights the critical role of inflammation and its markers, such as NLR and IL-6, in endothelial health and potential cardiovascular outcomes. Also, IL-8 and MCP can be used in further studies to evaluate their correlation with endothelial dysfunction.

## 5. Conclusions

Endothelial dysfunction secondary to COVID-19 infection represents a significant cardiovascular risk, regardless of the infection’s severity. Detecting markers of endothelial dysfunction that are easy to determine and cost-effective is crucial, particularly in the current context, as COVID-19 is no longer in the public health spotlight. Assessing endothelial dysfunction in all COVID-19 patients is essential, as its presence necessitates the implementation of therapeutic strategies aimed at minimizing vascular damage.

In this study, an elevated NLR demonstrated a similar correlation with reduced FMD, a marker of endothelial dysfunction, to that found in other inflammatory markers, such as IL-6. This finding supports the potential utility of the NLR as a diagnostic marker for endothelial dysfunction, complementing existing inflammatory markers and improving diagnostic accuracy.

These results emphasize the importance of incorporating routine evaluation of endothelial function in clinical practice, particularly for post-COVID-19 patients, to mitigate long-term cardiovascular risks.

## Figures and Tables

**Table 1:** Mean values of analyzed parameters compared between the two groups.

	Group 1 (N = 99)			Group 2 (N = 99)			*p*	95% CI
	Mean Value	95% CI	SD	Mean Value	95% CI	SD		
Age	63.78	61.69 to 66.38	13.01	62.65	60.29 to 65.01	11.82	0.5227	−4.61 to 2.35
ESR	38.06	32.92 to 43.19	25.75	16.18	13.95 to 18.41	11.17	<0.0001	−27.45 to −16.29
Fibrinogen	507.71	474.83 to 540.59	164.86	337.58	319.83 to 355.33	89.00	<0.0001	−207.33 to −132.92
CRP	48.40	36.46 to 60.34	59.86	7.22	5.74 to 8.71	7.44	<0.0001	−53.20 to −29.15
D-Dimeri	1.23	1.04 to 1.42	0.96	0.55	0.50 to 0.60	0.25	<0.0001	−0.87 to −0.48
Leucytes	5.97	5.46 to 6.47	2.53	7.72	7.33 to 8.11	1.95	<0.0001	1.11 to 2.39
Granulocytes	4.12	3.65 to 4.60	2.38	5.35	5.05 to 5.64	1.47	<0.0001	0.67 to 1.78
NLR	4.74	3.70 to 5.78	5.20	3.14	2.82 to 3.45	1.58	0.0038	2.68 to 0.52
Platelet count	237.32	182.05 to 292.59	277.11	241.53	215.64 to 267.42	129.82	0.8913	−56.59 to 65.02
Hb	13.10	12.81 to 13.40	1.47	13.29	13.05 to 13.52	1.17	0.3266	−0.18 to 0.56
Ht	39.04	38.19 to 39.90	4.29	38.75	38.11 to 39.40	3.23	0.5883	−1.35 to 0.77
FMD	7.72	7.52 to 7.92	0.99	13.72	13.35 to 14.09	1.83	<0.0001	5.58 to 6.41
TC	206.02	198.09 to 213.95	39.76	193.66	187.52 to 199.80	30.77	0.0154	−22.32 to −2.38
HDLc	41.56	40.27 to 42.85	6.46	43.16	42.04 to 44.27	5.59	0.0647	−0.098 to 3.29
TC/HDLc	5.18	4.85 to 5.51	1.67	4.61	4.38 to 4.85	1.18	0.0068	−0.97 to −0.15

N—number of participants, CI—confidence interval, SD—standard deviation, ESR—erythrocyte sedimentation rate, CRP—C-reactive protein, IL—interleukin, Hb—hemoglobin, Ht—hematocrit, NLR—neutrophil/lymphocyte ratio, FMD—flow-mediated diameter, TC—total cholesterol, HDLc—high-density lipoprotein cholesterol, TC/HDLc—total cholesterol/high-density lipoprotein cholesterol ratio. Group 1—post-COVID-19 patients, Group 2—patients without a COVID-19 history. An analysis of the data presented in Table 1 reveals that patients in the post-COVID-19 group (Group 1) exhibited significantly elevated mean values for all inflammatory markers evaluated. The increases in erythrocyte sedimentation rate, fibrinogen, C-reactive protein, leukocyte count, and granulocyte count were all statistically significant (*p* < 0.0001). Additionally, a significant increase in D-dimer levels (*p* < 0.0001) was observed despite normal platelet counts. Mean hemoglobin and hematocrit levels showed no significant differences between the two groups. Total cholesterol levels were significantly higher in Group 1 (*p* = 0.0154), with an elevated total cholesterol-to-HDL cholesterol (HDLc) ratio (*p* = 0.0068) despite similar mean HDLc levels between groups.

**Table 2:** Correlation of evaluated parameters with FMD in post-COVID-19 patients (Group 1).

	Correlation Coefficient r	*p*	95% CI for r
PGGI	−0.6164	<0.0001	−0.7255 to −0.4771
ESR	−0.7935	<0.0001	−0.8567 to −0.7068
Fibrinogen	−0.6968	<0.0001	−0.7861 to −0.5790
CRP	−0.6488	<0.0001	−0.7502 to −0.5177
LDH	−0.4312	<0.0001	−0.5793 to −0.2555
D-Dimer	−0.5773	<0.0001	−0.6955 to −0.4288
Ferritin	−0.5237	<0.0001	−0.6535 to −0.3639
IL-6	−0.5844	<0.0001	−0.7010 to −0.4375
Leucocyte	−0.3614	0.0002	−0.5216 to −0.1766
Granulocyte	−0.4896	<0.0001	−0.6265 to −0.3235
Thrombocyte	−0.02776	0.7851	−0.2239 to 0.1706
Hb	0.1721	0.0884	−0.02617 to 0.3574
Ht	0.1423	0.1600	−0.05670 to 0.3304
NLR	−0.4800	<0.0001	−0.6188 to −0.3121

*p*—statistical significance coefficient, CI—confidence interval, PGGI—pulmonary “groud-glass” imaging on pulmonary computed-tomography, ESR—erythrocyte sedimentation rate, CRP—C-reactive protein, IL—interleukin, Hb—hemoglobin, Ht—hematocrit, NLR—neutrophil/lymphocyte ratio.

**Table 3:** Correlation of evaluated parameters with FMD in patients without a history of COVID-19 (Group 2).

	Correlation Coefficient r	*p*	95% CI for r
ESR	−0.7204	<0.0001	−0.8036 to −0.6097
Fibrinogen	−0.4957	<0.0001	−0.6314 to −0.3307
CRP	−0.6240	<0.0001	−0.7313 to −0.4866
D-Dimer	−0.5100	<0.0001	−0.6427 to −0.3476
Leucocyte	0.05938	*p* = 0.5593	−0.1397 to 0.2538
Thrombocyte count	0.002875	*p* = 0.9775	−0.1947 to 0.2002
Hb	−0.2081	*p* = 0.0387	−0.3895 to −0.01118
Ht	−0.1208	*p* = 0.2335	−0.3108 to 0.07846
NLR	0.1030	*p* = 0.3102	−0.09635 to 0.2945

*p*—statistical significance coefficient, CI—confidence interval, ESR—erythrocyte sedimentation rate, CRP—C-reactive protein, IL—interleukin, Hb—hemoglobin, Ht—hematocrit, NLR—neutrophil/lymphocyte ratio.

**Table 4:** Simple linear regression analysis of factors influencing FMD in post-COVID-19 patients (Group 1).

	R^2^	Intercept	95% CI	Slope	95% CI	*p*
PGGI	0.3800	8.2338	8.0293 to 8.4384	−0.05324	−0.06694 to −0.03953	<0.0001
ESR	0.6297	8.8979	8.6798 to 9.1160	−0.03076	−0.03551 to −0.02600	<0.0001
Fibrinogen	0.4855	9.8691	9.4022 to 10.3360	−0.004219	−0.005094 to −0.003343	<0.0001
CRP	0.4210	8.2509	8.0547 to 8.4472	−0.01082	−0.01338 to −0.008262	<0.0001
LDH	0.1859	8.6063	8.1940 to 9.0186	−0.003309	−0.004705 to −0.001914	<0.0001
D-Dimer	0.3333	8.4639	8.1978 to 8.7299	−0.5974	−0.7677 to −0.4272	<0.0001
Ferritin	0.2742	8.2468	8.0058 to 8.4877	−0.0009246	−0.001228 to −0.0006215	<0.0001
IL-6	0.3416	8.0852	7.8944 to 8.2759	−0.01398	−0.01789 to −0.01007	<0.0001
Leucocyte	0.1306	8.5755	8.0966 to 9.0545	−0.1421	−0.2159 to −0.06820	=0.0002
Granulocyte	0.2397	8.5739	8.2235 to 8.9243	−0.2052	−0.2788 to −0.1315	<0.0001
NLR	0.2304	8.1640	7.9259 to 8.4021	−0.09208	−0.1260 to −0.05817	<0.0001

R^2^—coefficient of determination, CI—confidence interval, *p*—statistical significance coefficient, PGGI—pulmonary “groud-glass” imaging on pulmonary computed-tomography, ESR—erythrocyte sedimentation rate, CRP—C-reactive protein, IL—interleukin, NLR—neutrophil/lymphocyte ratio.

**Table 5:** Simple linear regression analysis of factors influencing FMD in patients without a history of COVID-19 (Group 2).

	R^2^	Intercept	95% CI	Slope	95% CI	*p*
ESR	0.5190	15.6438	15.1924 to 16.0952	−0.1185	−0.1415 to −0.09553	<0.0001
Fibrinogen	0.2457	17.1826	15.9209 to 18.4443	0.01024	−0.01385 to −0.006624	<0.0001
CRP	0.3894	14.8397	14.4373 to 15.2422	−0.1541	−0.1930 to −0.1152	<0.0001
D-Dimer	0.2601	15.7651	15.0030 to 16.5273	−3.6804	−4.9313 to −2.4296	<0.0001
Leucocyte	0.003526	13.2952	11.7906 to 14.7998	0.05574	−0.1331 to 0.2446	=0.5593
Granulocyte	0.002672	13.3810	11.9885 to 14.7734	−0.1865 to 0.3153	−0.1865 to 0.3153	=0.6114
NLR	0.01062	13.3509	12.5344 to 14.1674	0.1194	−0.1129 to 0.3517	=0.3102

R^2^—coefficient of determination, CI—confidence interval, *p*—statistical significance coefficient, ESR—erythrocyte sedimentation rate, CRP—C-reactive protein, NLR—neutrophil/lymphocyte ratio.

**Table 6:** Multiple linear regression analysis of factors affecting FMD in post-COVID-19 patients (Group 1).

Method	Enter					
Sample size	99					
Coefficient of determination R^2^	0.6490					
R^2^-adjusted	0.6261					
Multiple correlation coefficient	0.8056					
Residual standard deviation	0.6103					
Regression equation						
Independent Variables	Coefficient	Std. Error	t	*p*	r_partial_	r_semi partial_
Constant	9.1606					
Fibrinogen	−0.0004625	0.007784	−0.594	0.5538	−0.06183	0.03670
ESR	−0.02656	0.005553	−4.784	<0.0001	−0.4463	0.2955
IL-6	0.001011	0.002282	0.443	0.6586	0.04616	0.02738
NLR	−0.02871	0.01441	−1.993	0.0492	−0.2034	0.1231
CRP	0.0004013	0.001937	0.207	0.8364	0.02159	0.01279
LDH	−0.0003633	0.0005576	−0.652	0.5163	−0.06777	0.04024
Significance level	*p* < 0.0001					

*p*—statistical significance coefficient, ESR—erythrocyte sedimentation rate, CRP—C-reactive protein, IL—interleukin, LDH—lactate-dehydrogenase, NLR—neutrophil/lymphocyte ratio.

**Table 7:** ANOVA test for quartiles of the neutrophil-to-lymphocyte ratio (NLR) and IL-6 in each group (IL-6: interleukin-6, NLR: neutrophil-to-lymphocyte ratio).

Quartile	Mean IL-6 (pg/mL)	Mean NLR	Sample Size (n)	Standard Deviation (SD)
Q1	8.07	-	25	7.13
Q2	12.83	-	25	16.34
Q3	40.58	-	24	64.23
Q4	41.53	-	25	43.14
ANOVA *p*-value	0.003	-	-	-

## Data Availability

Data will be made available on valid written request addressed to the corresponding author.
